# Is it possible to stage schizophrenia? A systematic review

**DOI:** 10.1038/s41398-022-01889-y

**Published:** 2022-05-11

**Authors:** Clara Martínez-Cao, Lorena de la Fuente-Tomás, Ainoa García-Fernández, Leticia González-Blanco, Pilar A. Sáiz, María Paz Garcia-Portilla, Julio Bobes

**Affiliations:** 1 Department of Psychiatry, Universidad de Oviedo, Oviedo, Spain; 2 Instituto de Investigación Sanitaria del Principado de Asturias (ISPA), Oviedo, Spain; 3 Instituto Universitario de Neurociencias del Principado de Asturias (INEUROPA), Oviedo, Spain; 4 Centro de Investigación Biomédica en Red de Salud Mental (CIBERSAM), Madrid, Spain; 5 Servicio de Salud del Principado de Asturias (SESPA), Oviedo, Spain

**Keywords:** Psychiatric disorders, Schizophrenia

## Abstract

**Introduction:**

A staging model is a clinical tool used to define the development of a disease over time. In schizophrenia, authors have proposed different theoretical staging models of increasing complexity. Therefore, the aims of our study were to provide an updated and critical view of the proposed clinical staging models for schizophrenia and to review the empirical data that support them.

**Methods:**

Systematic literature review following PRISMA guidelines. From the PubMed database and backward reference search, a total of 141 records were retrieved, but only 20 were selected according to the inclusion criteria: (a) available in English; (b) participants with schizophrenia ≥ 18 years; and (c) theoretical and empirical research studies intended to develop, validate, and/or improve staging models of schizophrenia.

**Results:**

Different clinical staging models for schizophrenia were identified, information about the proposed stages was tabulated and presented in the Results section (Tables 1, 2). Most of which include neuroimaging, functioning, and psychopathology, but only two models add objective biomarkers and none include patient point of view. However, few models have been psychometrically tested or used small samples and thus have been validated only partially. In addition, five studies proposed therapeutic interventions according to the stage of the disorder from a theoretical point of view.

**Discussion:**

In conclusion, it is possible to stage schizophrenia, but the models developed have several limitations. Empirical validation and inclusion of more specific biomarkers and measures of other life areas affected by schizophrenia could help in the development of more valid models.

## Introduction

A staging model is a clinical tool used to define the development of a disease over time [1] that allows for integration of clinical information together with biomarkers, comorbid disorders, and other relevant variables, thus promoting personalized interventions [2]. These models have acquired primary importance in different areas of medicine, such as oncology and cardiology.

Due to the lack of studies that treat psychotic and affective disorders as developmental diseases, Fava and Kellner [3] developed the first clinical staging model in psychiatry. Since then, there has been increasing interest in clinical staging models for severe mental disorders, especially for psychotic disorders, in order to distinguish earlier, nonspecific phases of illness from later and more severe features associated with chronic disease [4]. Staging models provide clinicians a selection of treatments adapted to the early stages of the disease to prevent progression and provide remission. Furthermore, they may offer a unitary framework and individualization of care, which minimize heterogeneity in clinical practice and improve patient prognosis [5, 6].

The first staging model for schizophrenia was developed in 1993 [3]. Since then, authors have proposed different theoretical staging models of increasing complexity. These models are based on different dimensions that are affected by the progression of the disorder. However, although different staging models have been proposed, there is no consensus, nor is there enough empirical evidence to support the use of these models in clinical practice. Furthermore, reviews on the topic have focused on the biological basis of the disease, neglecting its multidimensional nature [1, 6–9]. Therefore, taking a multidimensional perspective, the first aim of this systematic literature review is to provide a global updated and critical view of the clinical staging models proposed for schizophrenia. The second aim is to review the empirical data supporting these models, and ultimately, the biological and psychological interventions proposed according to the stages.

## Method

The present systematic review follows Preferred Reporting Items for Systematic Reviews and Meta-Analyses (PRISMA) guidelines [10] (Supplementary Table 1). However, we did not prepare a protocol for this review, nor was it registered.

### Search strategy

For this review, we conducted a systematic search in the PubMed database. In order to limit the results to the most relevant, the search strategy was: (“staging”) AND (“schizophrenia”). We supplemented the database search by reviewing reference lists of articles meeting our inclusion criteria (backward reference search).

### Study selection

The articles found were examined in order to select those that met the following inclusion criteria:

- Available in English.
- Participants with schizophrenia ≥ 18 years.
- Theoretical and empirical research studies intended to develop, validate, and/or improve staging models of schizophrenia (reviews were therefore excluded).

**Fig. 1:**
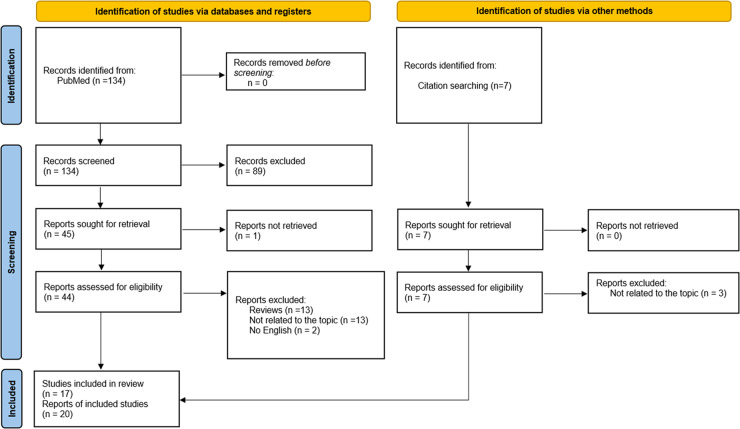
Identification of studies for inclusion in systematic review.

The database searches (completed July 20, 2021) returned 134 records (see Fig. 1 for full flowchart). One researcher (CMC) reviewed all record titles and abstracts. Then that researcher screened the full text of the articles for inclusion. If in doubt, two additional researchers were consulted (M.P.G.P., L.F.T.). After identifying 45 full-text reports, one report was not accessible (we tried to contact the authors but did not receive a response [11]), 26 were excluded because they were reviews (*n* = 13) or did not focus on a clinical staging model for schizophrenia (*n* = 13), and two articles were not available in English. Later, we searched for articles cited in any of the included studies and found seven articles potentially fulfilling the inclusion criteria. Three reports were excluded because they did not focus on a clinical staging model for schizophrenia; thus, four met the inclusion criteria. Therefore, of the identified records (*n* = 141), 20 reports on 17 studies constitute the final sample used in this review.

### Data extraction

Data were extracted from the studies using the data extraction form (Tables 1, 2, 3) and were collected by one researcher (C.M.C.). If in doubt or in case of unclear information, a consensus was reached after discussion with two of the other authors (M.P.G.P. and L.F.T.).

**Table 1:** (a, b) Staging models for schizophrenia.

(a) *Staging models for schizophrenia*								
	Fava and Keller [3]	Cosci and Fava [12]	Lieberman et al. [13]	Singh et al. [14]	Agius et al. [7]	McGorry et al. (2010)	Hickie et al. [17]	Godin et al. [18]
**Stage 0**			**Premorbid phase** Mild physical abnormalities Mild cognitive impairments Social deficits Neurodevelopmental abnormalities			**Premorbid phase** No symptoms Increased risk of psychotic or mood disorders		
**Stage 1**	**Prodromal phase** Affective and negative symptoms Deterioration of functioning	**Prodromal phase** Deterioration of functioning	**Prodromal phase** Nonspecific mood symptoms Neuroplastic dysfunction	**Prodromal phase** Nonspecific disturbance of mood, thinking, behavior, perception, and functioning	**Prodromal phase** Loss of gray matter	**Prodromal phase**	**Prodromal phase**	
Stage 1a				(P1) Unease		Nonspecific symptoms Neurocognitive deficits/severe mood disorders Functional changes	Distress disorder History of developmental or learning disorder GAF/SOFAS>70–100 QIDS: 0–11 Reductions in total sleep	
Stage 1b				(P2) Non-diagnostic symptoms		Moderate symptoms Moderate cognitive changes Functional impairment (GAF 70)	Attenuated syndrome GAF/SOFAS: 60–70 QIDS: 11–20 YMRS>9 Mild deficits in executive functions Emerging gray matter loss Pro-inflammatory cytokine elevation (among others)	
**Stage 2**	**First psychotic episode (DM-III-R)**	**First psychotic episode**	**First psychotic episode** Cognitive impairment, negative symptoms, social deficits Neurochemical sensitization involving meso-limbic-cortical-striatal circuits	**First psychotic symptoms**	**First psychotic episode**	**First psychotic symptoms** Neurocognitive deficits Functional impairment (GAF 30–50)	**Discrete disorder** GAF/SOFAS: 40–60 QIDS>20 YMRS>15 Moderate deficits in executive functions Significant loss of gray matter Pro-inflammatory cytokine elevation with tendency towards reduced markers of cell-mediated immunity (among others)	
Stage 2a								Clinical full remission and good functioning
Stage 2b								Mild psychotic symptomatology Mildly impaired functioning
**Stage 3**	**Residual phase (DSM-III-R)**	**Residual phase**	**Chronic phase** Psychosis Neurotoxicity, loss of cell processes and apoptosis	**Diagnosis** Delusions, hallucinations, first rank symptoms, thought disorder or catatonic symptoms	**Chronic phase** Cortical and subcortical brain areas affected		**Recurrent/ persistent disorder** GAF/SOFAS<40 Severe reductions in executive functions and/or social cognition Reduction in hippocampal volume	
Stage 3a						Partial remission from the first episode		Incomplete remission Moderate level of functioning
Stage 3b						Remission		Severely ill patients Severe impairment in functioning
Stage 3c						Multiple relapses		
**Stage 4**	**Subchronic phase** (6–24 months)	**Chronic phase**				**Chronic phase** Severe, persistent and unremitting illness	**Chronic phase** GAF/SOFAS<30 Enlarged ventricles	Severe psychotic symptomatology Highly impaired in functioning Depressive symptoms
**Stage 5**	**Chronic phase** (>24 months)							

*(b) Staging models for schizoprenia*			
	Dragioti et al. (2016)	Fountoulakis et al. [20]	Fountoulakis et al. [21]
**Stage 1**	**Early stage** **(18–34 years of age)** PANSS domains: Negative domain Depression-Anxiety Hostility-Aggression Disorganization Positive domain Delusional hostility domain Depression domain	**First 3 years of evolution** Positive symptoms dominant Negative, anxiety and depressive symptoms stable Excitement and hostility increase	**First 3 years of evolution** Positive symptoms
**Stage 2**	**Middle stage** **(35–44 years of age)** PANSS domains: Negative domain Positive domain Hostility-Aggression Depression domain Disorganization Residual negative disorganization	**3–12 years of evolution** Excitement and hostility dominant Positive symptoms stable Negative, anxiety, depressive symptoms, and neurocognitive deficit increase	**3–12 years of evolution** Excitement and hostility
Stage 2a		**3–6 years of evolution** Positive symptoms tend to remit Excitement and hostility increase Negative, anxiety and depressive symptoms increase	
Stage 2b		**6–12 years of evolution** Excitement and hostility stable Positive symptoms stable Negative, anxiety and depressive symptoms increase Neurocognitive deficit rise	
**Stage 3**	**Advanced stage** **(≥45 years of age)** PANSS domains: Negative domain Positive domain Hostility-Aggression Depression-Anxiety Disorganization Neurocognitive disorder Residual domain	**12–25 years of evolution** Negative symptoms and neurocognitive deficit rise Depression and anxiety dominant	**12–25 years of evolution** Depressive and anxiety symptoms
Stage 3a		**12–18 years of evolution** Excitement and hostility dominant Negative and positive symptoms increase Neurocognitive deficits increase	
Stage 3b		**18–25 years of evolution** Negative symptoms and neurocognitive deficit dominant Depression and anxiety dominant Excitement and hostility decrease Positive symptoms decrease	
**Stage 4**		**25-≥40 years of evolution** Neurocognitive deficits increase	**25-≥40 years of evolution** Neurocognitive impairments
Stage 4a		**25–40 years of evolution** Neurocognitive impairment dominant Negative symptoms increase Depression and anxiety decrease	
Stage 4b		**≥40 years of evolution** Neurocognitive deficits dominant Excitement and hostility increase	

*GAF* Global Assessment of Functioning, *SOFAS* Social and Occupational Functioning Scale, *QIDS* Quick Inventory of Depressive Symptomatology, *YMRS* Young Mania Rating Scale.

**Table 2:** Validation of the clinical staging models.

		Objective vs Validation/Feasibility	Sample	Stages	Conclusions
**McGorry et al. (2010)**	Berendensen et al. (2018)	To examine the construct validity of the staging model by measuring differences in severity of clinical profiles and therapeutic improvement between clinical stages.	*n* = 258	Stage 2 = 48 Stage 3b = 100 Stage 3c = 81 Stage 4 = 29	Only stages 3c and 4 showed adequate construct validity [significant differences were found for negative symptoms (*F* = 4.56, *p* < 0.010), number of psychotic episodes (*F* = 13.65, *p* < 0.010), and premorbid functioning (*F* = 7.33, *p* < 0.001) according to stages].
	Berendensen et al. (2019)	To determine the inter-rater reliability of the clinical staging. To investigate whether a short course can improve reliability.	*n* = 114 (no training)	Stage 2 = 22 Stage 3a = 1 Stage 3b = 39 Stage 3c = 41 Stage 4 = 11	The inter-rater reliability in clinical staging was better after training (*ICC* = 0.57 vs ICC = 0.75).
			*n* = 100 (with training)	Stage 2 = 22 Stage 3a = 1 Stage 3b = 50 Stage 3c = 22 Stage 4 = 5	
	Godin et al. [18]	To classify patients according to the model. To use clinical, cognitive, and treatment variables to explore validity. To explore the stability of the model.	*n* = 770	Stage 2a = 89 Stage 2b = 272 Stage 3a = 241 Stage 3b = 112 Stage 4 = 56	Follow-up at one year showed good stability (62% of the sample remained stable).
	Berendensen et al. (2021)	To examine differences in severity for dimensional symptoms of psychosis between stages.	*n* = 291	Stage 2 = 62 Stage 3a = 9 Stage 3b = 127 Stage 2b = 75 Stage 4 = 18	Significant differences in the severity of symptoms only were found in stages 3c and 4 [hallucinations (*H* = 14.34, *p* = 0.006), negative symptoms (*H* = 19.67, *p* = 0.001), and cognitive deficits (*H* = 26.29, *p* < 0.001)].
**Hickie et al**. [17]	Hickie et al. [17]	To demonstrate the inter-rater reliability of the model.	*n* = 209	Stage 1a = 21 Stage 1b = 112 Stage 2 = 53	The inter-rater reliability was acceptable (*K* = 0.72, *p* < 0.001).
	Romanowska et al. [25]	To assess neurocognition in a sample of patients in the first stages of schizophrenia.	*n* = 243	Stage 0 = 41 Stage 1a = 52 Stage 1b = 108 Controls = 42	Patients in stage 1b presented significantly poorer cognitive performance (MATRICS Overall Composite *F* = 5.70, *p* < 0.001).
	Addington et al. [26, 27]	To identify sample that met different stages of risk for the development of a serious mental illness (SMI) based on a published clinical staging model. To determine whether participants allocated to the different stages were a good fit to the model.	*n* = 243	Stage 0 = 41 Stage 1a = 52 Stage 1b = 108 Controls = 42	Patients in stage 1b had significantly more severe symptoms than participants in lower stages [functioning (*F* = 77.10, *p* < 0.002), depressive symptoms (*F* = 30.10, *p* < 0.002), and prodromal psychotic symptoms (*F* = 37.30, *p* < 0.002)].
	Addington et al. [28]	To describe changes in participants over 12 months to understand the course of illness progression in its earliest stages.	*n* = 243	Stage 0 = 41 Stage 1a = 53 Stage 1b = 107 Controls = 42	Follow-up at one year showed stability (only 7–9% of the participants changed stage in the follow-up).

*ICC* Intraclass Correlation Coefficient, *K* Kappa Statistics, *MATRICS* The Measurement and Treatment Research to Improve Cognition in Schizophrenia.

**Table 3:** Interventions proposed by stages.

	Lieberman et al. [13]	Agius et al. [7]	McGorry et al. (2010)	Cornblatt [33]	Carrion et al. [36]
**Stage 0**	Potential for gene therapy		Psychoeducation		
Stage 0a				Psychotherapy	Antidepressant
Stage 0b				Antipsychotics Antidepressant Psychotherapy	Antipsychotics Antidepressant
Stage 0c				Antipsychotics Antidepressant Psychotherapy	
**Stage 1**	Atypical antipsychotics Stress reduction therapy	Antipsychotics Antidepressant Cognitive therapy	Family psychoeducation Substance abuse reduction	Antipsychotics Psychotherapy	
Stage 1a			Counseling and problem solving Exercise		
Stage 1b			CBT Cognitive intervention		
**Stage 2**	Antipsychotics	Optimize medication Psychosocial interventions^a^	Family psychoeducation CBT Substance abuse reduction Atypical antipsychotics Antidepressant/mood stabilizers Work rehabilitation		
**Stage 3**	Antipsychotics and potential experimental agents as adjunctive treatment	Optimize medication: clozapine Psychosocial interventions^a^ Relapse prevention			
Stage 3a			Medical strategies Psychosocial intervention		
Stage 3b			Relapse prevention		
Stage 3c					
**Stage 4**			Clozapine		

*CBT* cognitive behavioral therapy.

^a^Familiy interventions, Compliance therapy, Relapse intervention, Psychoeducation.

To explain the models developed to date, we collected the following data:

- Report: authors and year.
- Clinical staging models: including phases of the proposed models and characteristics of the phases (neuroimaging, functioning, psychopathology, cognition, affective symptoms, and endophenotypic and biological markers, if applicable).
- Interventions: including psychological and pharmacological interventions theoretically proposed by authors for each phase.
- Validation of staging models: including the objective of the research, sample sizes, stages of the participants, and conclusions of the studies.

## Results

### Description of studies

A total of 20 articles met the inclusion criteria for this systematic review as follows:

- Eight articles report a clinical staging model of schizophrenia (6 based on multidimensional domains and the other two based on a single domain).
- Seven articles validate any of these multidimensional models.
- Three articles validate and/or improve these models.
- And finally, two articles propose interventions for prodromal phases of schizophrenia from a theoretical point of view.

**Fig. 2:**
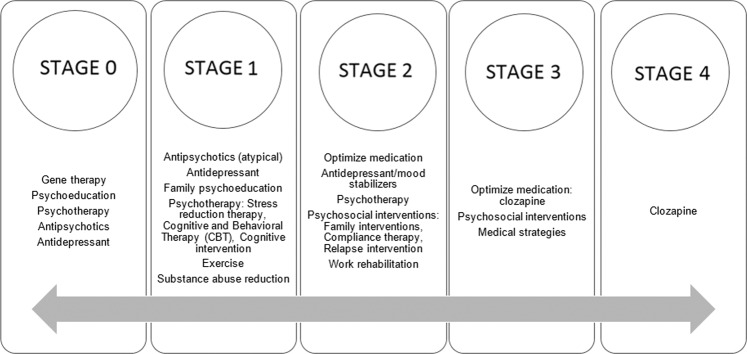
Interventions proposed by stages.

To clearly and transparently present our results, we created three tables (Tables 1, 2, and 3) and a figure (Fig. 2) that summarize and complete the information provided in this section.

### General staging models for schizophrenia

Fava and Kellner developed the first staging model for schizophrenia in 1993 [3]. They proposed a 5-stage model based on previous research findings on clinical progression of the mental disorder. In their model, the initial phases of the disease are differentiated according to patient functioning and psychopathological characteristics. For the later phases, only DSM-III-R criteria and length of illness are used. A few years later, with the aim of integrating later findings, Cosci and Fava [12] redefined the model, eliminating the subchronic phase, reducing the model to 4 phases (Table 1a).

In 2001, Lieberman et al. [13] developed a 4 phase-model comprising clinical features and underlying pathophysiological process. Specifically, the authors used physical anomalies, changes in neuroanatomy, and cognitive and social deficits as indicators of disease progression. Furthermore, their model differs from the previous one in that it includes a first stage termed “Premorbid,” characterized by physical, cognitive, and coordination problems.

Focusing on the prodromal phase, Singh et al. [14] divided their 3-stage model into 2 subphases: a first period of unease (P1) and a second period of non-diagnostic symptoms (P2). They describe unease as a concept similar to the “morbid unease” proposed by Copeland [15], where the symptom was definitely present, but not of a severity to reach a level of caseness. This model differentiates between the phases mainly according to clinical progression, so that the emergence of first psychotic symptoms (FPS) constitutes the second stage, and the development of symptoms leading to a definitive diagnosis constitutes the last stage. The Nottingham Onset Schedule (NOS) a short, guided interview to measure the onset of psychosis is based on this model.

Focusing on neuroanatomical changes based on previous cognitive and neuroimaging data, Agius et al. [7] proposed three stages: the prodrome, the first episode, and the chronic phase. These stages are based on the development of the disease and the progressive loss of gray matter, resulting in changes in patient cognition (Table 1a).

In 2010, McGorry et al. [16] completed the development of a complex model reflecting the clinical and biological progression of the disease, where stages are not static categories and patients can return to previous phases. For the first time, this model includes, in addition to neuroimaging, functioning, and psychopathology, cognition, affective symptoms, endophenotypic and biological markers. Regarding biomarkers, they proposed prepulse inhibition, P 50, smooth pursuit eye movements, olfactory deficits, Hypothalamic-Pituitary-Adrenal (HPA) dysregulation, niacin sensitivity, folate status, and oxidative stress as markers of illness state, trait, and progression. The initial stage corresponds to an increased risk of psychotic or mood disorders without symptoms. In stage 1, we can differentiate between patients with nonspecific symptoms (stage 1a) and patients with moderate psychotic symptoms and impaired functioning (stage 1b). Stage 2 corresponds to onset of the disease with severe psychotic symptoms. Stage 3 is divided into partial remission of the first episode (3a), a new episode (3b), and multiple relapses (3c). And finally, stage 4 is chronic, severe, and persistent illness. Based on this model, Hickie et al. [17] developed a similar classification eliminating the three subphases of the third stage and including the patient’s personal history. In their study, with the aim of assessing the feasibility of their model, they applied the staging model to young people with impaired functioning and mild symptoms of psychosis, anxiety, and/or depression. They proposed clinical features, neuropsychology, neuroimaging, and biological markers depending on the phases of the disease (Table 1a). However, unlike the McGorry et al. [16] model, it is not possible to return to previous stages. Furthermore, this model takes into account mainly three biomarkers: Firstly, an event-related potential (ERP), with a progressive influence from stage 1b to 4; secondly, an HPA dysfunction, which appears in stage 2; and finally, a neuroimmunological disorder characterized in the first stages by an increase in pro-inflammatory cytokines that leads to a reduction in cellular immunity in the later stages. A few years later, Godin et al. [18] suggested that the McGorry et al. [16] model could be improved by subdividing the intermediate stages (2 and 3) and by adding clinical elements such as mood symptoms and cognitive deficits (Table 1a). Their stages were characterized by a progressive deterioration of functioning and an increase in symptom severity, with a rise of depressive symptoms in the last phase.

Recently, different authors [19–21] have developed a staging model of schizophrenia based on patient PANSS scores (Table 1b). After performing an exploratory factor analysis using a principal component analysis, Dragioti et al. [19] developed a six-factor structure that differs among the stages of the disease based on patient age. In the first stage (18–34 years of age), negative and affective symptoms are predominant. However, in the second stage (35–44 years of age), positive and negative symptoms are factors that explain more variance. Finally, in the third stage (≥45 years of age), neurocognitive deficits and the residual domain rise.

Subsequently, Fountoulakis et al. [20] analyzed the predominant PANSS factors according to the length of the disorder using an exploratory factor and discriminant function analysis. They identified four phases. In the first stage (3 years of duration), positive symptoms predominate. In the second stage (3–12 years of duration), which is divided into two phases (2a, 2b), the dominant symptomatology is excitement and hostility, while positive symptoms remain stable. In addition, in both phases, there is an increase in negative and affective symptoms, while in the most severe phase (2b), neurocognitive deficits also increase. The third stage (12–25 years of duration) is divided into two phases: 3a, which is dominated by excitement and hostility, and 3b, where affective and deficit symptoms (negative and cognitive symptoms) become more prominent. The fourth stage (25 years of duration) is subdivided into two stages (4a, 4b). Although the dominant factor in both subphases is neurocognitive deficit, in the more severe stage (4b), there is also an increase in hostility and excitement, and in the less severe stage (4a), negative and affective symptoms. Therefore, in order to clarify the relationship between the symptoms in the stages according to PANSS clinical dimensions, Fountoulakis et al. [21] identified the predominant factors in each phase of the disease. In the first stages, positive symptoms, excitement, and hostility are the dominant factors. However, over the course of the illness, affective and neurocognitive symptoms acquire predominance. Finally, negative symptoms remain stable to some extent through the stages, with a mild increase in stages 3b, 4a, and 4b (Table 1b).

### Clinical validity of staging models of schizophrenia

In recent years, the number of studies aiming to validate these models have increased. Specifically, we found 9 articles that try to validate the McGorry et al. [16] and Hickie et al. [17] models (see Table 2).

The McGorry et al. [16] model was validated for the first time by Berendsen et al. [22]. They designed a cross-sectional study with 258 acute ward patients where participants were classified into stage 2, stage 3b-c, or stage 4. Their results show that the McGorry et al. [16] model has acceptable construct validity between earlier and more chronic stages of the disease, where the number of psychotic episodes, lower medication adherence, and more functional impairments were associated with higher stages. One year later, with the aim of determining the inter-rater reliability of the model, Berendsen et al. [23] developed a study where a sample of clinicians attended a practical training course in clinical staging. The results demonstrated that inter-rater reliability was acceptable after training; however, assessments of living situation, trauma, functioning, and social support earned low consistency scores. Godin et al. [18] also analyzed the validity of the model in a prospective cohort of 770 stable schizophrenia outpatients. The results showed that, one year later, the majority of the patients were in the same stage and greater improvements occurred in more severe stages (Table 2). Recently, Berendsen et al. [24] developed a study whose results support the clinical validation of this staging model. Patients showed significant differences in the severity of negative, positive, and cognitive symptoms between stages. Furthermore, these authors propose dividing stage 2 based on duration of untreated psychosis (2a < 1 year; 2b ≥ 1 year), which is clinically important for the severity of negative symptoms.

In addition to applying their clinical staging procedure to 209 young people, the objective of Hickie et al. [17] was to demonstrate the inter-rater reliability of their model. They thus compared the stages assigned by the original treating clinicians who used an initial protocol and by the independent research team that had access to the sample’s medical records. Their results show that inter-reliability was moderate; however, this concordance increased when clinicians used the detailed criteria of the model. A few years later, with the aim of demonstrating that neurocognitive deficits are important indicators of the risk of severe mental illness (SMI) and valid for identification purposes, Romanowska et al. [25] described the neurocognitive functioning of 243 young people who met the risk criteria for SMI or who exhibited symptoms according to the early stages (0-1b) of the Hickie et al. [17] model. They found that neurocognitive performance was poorer in stage 1b with lower scores in speed of processing, attention, memory, and cognitive flexibility. In 2018, Addington et al. [26] described a study whose aim was to develop and validate an algorithm using the Hickie et al. [17] model. One year later, Addington et al. [27] compared clinical and sociodemographic information on patients in the first stages of the model. They found that the participants in stage 0 were similar to healthy controls, so they proposed discriminate patients with SMI risk be assessed for resilience in comparison with healthy subjects [27]. Recently, they analyzed the changes in this sample one year later [28]. The results show that changes in stages 0 and 1a were minimal; however, participants in stage 1b had the greatest improvement.

### Potential interventions according to clinical stages

In addition, to design a staging model for schizophrenia, some authors have proposed personalized interventions according to the stage of the disorder. These treatments could help prevent progression of the disorder and improve the patient’s prognosis (Table 3, Fig. 2).

In the premorbid phase, Lieberman et al. [13] proposed that gene therapy could be a potential treatment. In this sense, although recent results from Copy Number Variants (CNV) [29, 30] and Polygenic Risk Score (PRS) [31, 32] support the contribution of genetics to both schizophrenia and the transition from the ultra-high-risk state to psychosis, there is still no consensus on its clinical use. On the other hand, Cornblatt [33] analyzed preliminary findings from the Hillside Recognition and Prevention (RAP) program. In this program, patients were classified into four groups according to the severity of the symptoms. The first group with premorbid symptoms—the clinical high risk (CHR) group—received psychotherapy only, as in the early stages it is advisable to use less invasive treatment than at later stages. Finally, the worldwide effort made by the International Early Psychosis Association (IEPA) merits special attention. The IEPA was created in 1998 (currently called IEPA Early Intervention in Mental Health), with the aim of increasing knowledge related to the early phases of psychiatric disorders, their causes, and possible prevention strategies [34]. It has promoted the creation of early intervention units in different countries [35] and constitutes an international network that facilitates communication and collaboration among mental health professionals around the world [34].

For stage 1, antipsychotics have been proposed by different authors [6, 13, 33]. Cornblatt [33] and Carrion et al. [36] also found that treatment with antidepressants may be effective at reducing nonspecific symptom progression. Furthermore, environmental factors such as substance abuse and stress, associated with the onset of the disorder, are therapeutic targets [13, 16]. The authors have proposed psychological interventions such as cognitive, supportive, and the cognitive behavioral therapy (CBT) that can buffer risk and reduce progression to psychotic symptoms [16].

In stage 2, where clear psychotic symptoms are present and functioning is affected, pharmacological and psychosocial treatments are useful in order to stimulate functional and clinical recovery. Different authors have reported that atypical antipsychotics have shown better tolerability and can be more useful in these early stages [16]. Family is essential in providing care and support for the patients. These have been associated with fewer relapses, so the inclusion of family support therapies can be used to improve the patient’s prognosis [7, 16].

In later stages, medication adjustment and more aggressive treatments are chosen [7, 13, 16]. However, psychosocial interventions are still necessary as they can help prevent the risk of future relapses and the development of disability [7, 16].

## Discussion

We did a systematic review of staging models for schizophrenia. Over the years, more comprehensive general theoretical models have been developed and studies trying to validate these models have been performed. Over time, models have been improved by including biomarkers in addition to clinical, cognitive, and functional variables, giving them the true characteristic of staging models with objective data. However, to date there are still few models that include objective variables e.g., biomarkers, diagnosed physical comorbidities, or subjective self-reported variables, such as quality of life.

Biomarker research has confirmed that schizophrenia is a disease with chronic low-grade systemic inflammation [37–40], as well as cognitive impairment [41]. Although there are divergent results, different studies of inflammation seem to indicate a role of interleukins [42], specifically IL-6 and TNF-α, in clinical manifestations of the disease, and they are the most replicated in previous research [39]. Recent studies have also reported an association of IL-6 and TNF-α with negative symptoms [43]. For example, González-Blanco et al. [44] showed that interleukin IL-1β was associated with global symptomatology and IL-2 with anhedonia and avolition. The previous literature also reflects an association between schizophrenia and interleukins. A systematic review by Ribeiro-Santos, Teixeira and Vinicius [45] found that MCP-1 and IL-18 levels were associated with cognitive impairments in schizophrenia. In addition, Lim et al. [32], Perkins et al. [46], and He et al. [47] reported PRS as a potential biomarker for early cognitive deficits or for the transition from the ultra-high-risk state to established psychosis.

The frequent physical comorbidities in these disorders, such as cardiovascular diseases, diabetes, metabolic syndrome, etc., even in first episode patients [48, 49], have not been taken into account in the reviewed models. The scientific literature has also indicated the significant effect that physical illness has on the treatment and outcome of schizophrenia [50]. Considering that people with schizophrenia have 15 years’ lower life expectancy due to their physical health [51] and in view of the negative effects these diseases have on cognition and functioning [52, 53], physical comorbidities should also be taken into account when developing staging models in future studies.

It is of note that none of the reviewed models include patient-reported outcomes. It would be interesting to introduce the patient’s point of view into the stages. Obtaining information from patients themselves is of great value and should be considered complementary to the clinician’s point of view. Negative symptoms of schizophrenia involve internal experience and, therefore, are more accessible and suitable for self-reporting [54]. Furthermore, it is well known that quality of life is a distal marker of the results of disease interventions, which can only be reported by the patient. Thus, the effect of the disorder and its treatments on the life of patients should be taken into account in the different stages of the models.

Regarding validation, it is encouraging to see that there are increasing numbers of empirical clinical studies concerned with establishing the validity and reliability of the proposed theoretical models. Unfortunately, such studies have included small samples or patients who are in specific stages of the models. In this sense, Berendsen et al. [23] point out that the problem is mainly in the early stages of the disease. Therefore, their results apply only to specific stages of schizophrenia spectrum disorders and not to the preclinical stages of disease; this is because these patients were not included in the study as they had not been admitted to the hospital. Patients in premorbid and prodromal phases are not seen in clinical and hospital settings, making it difficult to access them. Thus, further high-quality studies are needed to empirically validate all the phases of these theoretical models.

For the purpose of increasing the utility of clinical staging models in daily clinical practice, therapeutic strategies have been proposed based on the disease stage. Biological and/or psychological interventions could thus be adjusted depending on the stage of disease. In the early stages, milder treatments can be effective, avoiding side effects and complications associated with unnecessarily high-intensity interventions [55]. We have reviewed different interventions proposed for each stage that could represent an advance in standardizing clinical practice and implementing personalized medicine, thus providing each patient with the most appropriate treatment depending on his/her disease stage. However, further research would be needed to confirm these therapeutic proposals.

This review has some methodological limitations. First, a limited number of databases were searched, and some relevant studies may be missing. However, this database is the most powerful for clinical research. Second, the different study methods greatly hinder the comparability of the data. The samples used were diverse in nature (i.e., age, context, length of illness, etc.). Finally, we found few longitudinal studies that report how patients move through the model; studies with follow-up were minimal and with short follow-up periods. Despite these limitations, it should be pointed out that, on the one hand, there is little tradition of developing clinical staging models in psychiatry and, on the other hand, we followed the PRISMA guidelines. Therefore, although not every study has been included, the methodology has been rigorous. Furthermore, to our knowledge, this is the first systematic review that uses a multidimensional perspective to provide an update on the clinical staging models of schizophrenia and the biological or psychological interventions proposed for each stage.

In conclusion, with this review, we have demonstrated that is possible to stratify schizophrenia. Psychiatrics have growing interest in clinical staging models for schizophrenia as evidenced by the increasing numbers of publications on the subject in recent years. However, these models would benefit from the inclusion of more specific and validated biomarkers and other measures of life areas affected by schizophrenia such as comorbidity with physical diseases and health-related quality of life. In addition, they need to be psychometrically tested before including them in daily clinical practice.

## Supplementary information

PRISMA_2020_Checklist
